# Subcritical Water Extraction of Chestnut Bark and Optimization of Process Parameters

**DOI:** 10.3390/molecules25122774

**Published:** 2020-06-16

**Authors:** Tanja Gagić, Željko Knez, Mojca Škerget

**Affiliations:** 1 Laboratory for Separation Processes and Product Design, Faculty of Chemistry and Chemical Engineering, University of Maribor, Smetanova 17, 2000 Maribor, Slovenia; tanja.gagic@um.si (T.G.); zeljko.knez@um.si (Ž.K.); 2 Faculty of Medicine, University of Maribor, Taborska ulica 8, 2000 Maribor, Slovenia

**Keywords:** subcritical water, sweet chestnut bark, ellagic acid, gallic acid, ellagitannins

## Abstract

The aim of the work was the optimization of the subcritical water extraction process of chestnut bark using Box–Behnken response surface methodology. The influence of process parameters, such as temperature, extraction time and solvent-solid ratio, on extraction yield, yield of the main compounds, total phenol content, total tannin content and antioxidant activity has been investigated. The identified compounds were ellagic and gallic acids, ellagitannins (vescalagin, castalagin, 1-*o*-galloyl castalagin, vescalin and castalin), sugars (maltose, glucose, fructose and arabinose) and sugar derivatives (5-HMF, furfural and levulinic acid). Finally, the optimal process conditions for obtaining the bark extract highly rich in ellagic acid and with satisfactory levels of total phenols and total tannins have been determined.

## 1. Introduction

Biomass represents the natural source of valuable components which could found application in many industries. The chestnut tree is the source of many important compounds [1]. Besides their seeds, used for fresh consumption and for preparation of chestnut purée, marron-glacé and chestnut flour [2,3,4], chestnut trees are also used for the construction of furniture, agronomic utensils, and cooperage [5]. Furthermore, chestnut trees are highly rich in phenolic compounds, especially in tannins which could find application in wine production, the leather industry and medicine [6]. Tannins are divided into two major groups: condensed and hydrolysable tannins. Condensed tannins, also known as proanthocyanidins, are oligomers or polymers composed of flavonoid units without sugar residues [7]. The condensed tannins were extracted from chestnut samples (leaves, catkins, seeds, bark and burs) and analyzed by Živković et al. [3]. Hydrolysable tannins are composed of esters of gallic acid or ellagic acid with a sugar core, mainly glucose, and thus are divided into two subclasses: gallotannins or ellagitannins, respectively [8]. The characteristic compounds of the sweet chestnut are ellagitannins, namely vescalagin, castalagin, vescalin and castalin [9]. Ellagitannins are complex derivatives of ellagic acid, which represent polyphenolic thermostable compounds which are slightly soluble in water, ether and alcohol [4]. Both ellagitannins and ellagic acid possess a number of health benefits and bioactivities, such as antioxidant, anticancer, anti-inflammatory, antibacterial, antimalarial, antiviral, cardioprotective and many others [4,10,11]. The content of biologically active compounds in the chestnut tree depends on type, location and climatic conditions where it grows, thus chestnuts from Portugal [5,12,13], Spain [14,15], Japan [16,17], Italy [2,18], Greece [19] were investigated. Furthermore, chestnut wood was investigated by Scalbert et al. [20] and Vivas et al. [21], but different parts of the chestnut were also studied: shell [2,5,13,14,15,22], bark [3,7,9,19,23], bur [3,24], leaves [3,25], nut [12], and fruits [19]. Chestnut fruits are rich in carbohydrates, mainly starch, but also contain proteins, fat, minerals and vitamins important for health [6]. The free sugars found in chestnut fruits are sucrose, glucose, fructose and maltose [6]. The bark and wood contain a much higher amount of phenols than the chestnut fruits [7]. Fuente-Maqueda et al. [26] determined the content of total phenols of 101.2 mg GAE/g, condensed tannins of 0.59 mg CE/g, gallotannins of 9.0 mg MG/g and ellagitannins of 6.0 mg EA/g in outer chestnut bark.

Different solvents were used for obtaining chestnut extracts. Vekiari et al. [19] used methanol for the extraction of chestnut bark and trifluoroacetic acid as a catalyst for the subsequent hydrolysis of extract and showed that hydrolyzed chestnut bark contains a considerably higher amount of ellagic acid than unhydrolyzed. They also proved that the outer part of the bark contains a higher amount of ellagic acid than the inner. Most barks produce polyphenols, especially proanthocyanidins, to protect trees against predators and pathogens which can cause decay [23]. Comandini et al. [9] performed the extraction of chestnut bark in methanol for 30 min at room temperature and then sonicated the sample in a water bath. Živković et al. [3] performed extraction of different parts of sweet chestnut (leaves, catkins, seed, bark and burs) by ultrasound using 50% ethanol. Chiarini et al. [7] extracted chestnut bark with methanol in order to study the cardiovascular effects of extracts. Besides ellagic acid and ellagitannins, the chestnut tree also contains gallic acid as one of the main compounds in its structure. Chestnut processing generates a waste product, which mainly includes shell, skin and bur. However, Vázquez et al. [14] investigated antioxidant activity and chemical composition of chestnut shell and eucalyptus bark. They showed that chestnut shell extracts possess higher antioxidant activity and amount of phenolics than eucalyptus bark. Vázquez et al. [14] used different extraction solvents for treating chestnut shells and the highest yield of extract was obtained using water as a medium (12.2%). The extraction was improved when 2.5% Na_2_SO_3_ was added into the water (yield of 25.62%). Their next study [15] showed that the extraction of chestnut shell gave the highest yield of 49.4% if 10% NaOH was added into the water. Vasconcelos et al. [5] used water, 70% methanol, 70% ethanol, 70% acetone and methylethylketone as extraction solvents for four Portuguese chestnut shell cultivars. The highest yield of total phenols, total condensed tannins and low molecular weight phenolics was obtained using 70% acetone at 20 °C. However, the investigations dealing with hydrothermal treatment of chestnut as an eco-friendly method are still scarce in the literature. Moure et al. [24] investigated the hydrolytic treatment of chestnut burs and it was shown that extracts with good bioactive properties were produced. Furthermore, the data on optimization of the extraction process of chestnut are also limited in the literature. Reinoso et al. [25] studied the optimization of antioxidants obtained by extraction of chestnut leaves using 96% ethanol, methanol and acidified water as extraction solvents, while Aires et al. [27] extraction and optimization of polyphenols, tannins and ellagitannins obtained from chestnut peels using water, Na_2_SO_3_ and NaOH at different concentrations of 1, 2, 4, and 8% in water. 

The aim of this paper was the subcritical water extraction of chestnut bark and the optimization of the process parameters. No comparable study on hydrothermal treatment of chestnut bark has been published before. The identified compounds were ellagic and gallic acids, ellagitannins (vescalagin, castalagin, 1-*o*-galloyl-castalin, vescalin, castalin), sugars (maltose, glucose, fructose and arabinose) and sugar derivatives (5-HMF, furfural and levulinic acid). In this study, the optimization of extraction yield, total phenol content, total tannin content, antioxidant activity and the yield of the main compounds was demonstrated. Finally, the optimal conditions for obtaining the extract with as high as possible content of phenolic compounds (total phenols and total tannins) and especially the content of ellagic acid were determined.

## 2. Results

### 2.1. Extraction Yield

Table 1 represents the obtained yield of extraction for the proposed experiments by the Box–Behnken method depending on three factors: temperature, time and solvent-solid ratio. The highest extraction yield was 44.9%, obtained at 150 °C, 10 min and for solvent-solid ratio of 20 mL/g. Observing the results, it can be concluded that yield decreases when increasing the temperature and time, because at higher temperatures and longer extraction times the hydrothermal degradation of water soluble compounds and their conversion to water insoluble products takes place. This is in agreement with the results of our previous work [1], where hydrolysis of chestnut tannins was studied and it was observed that secondary reactions of water-soluble fraction to char and gases were obvious at temperatures higher than 150 °C. For lower temperatures (150 and 200 °C), a higher solvent–solid ratio (30 mL/g) gives higher extraction yields, while at 250 °C, a higher yield was obtained for a lower solvent–solid ratio (10 mL/g). By using subcritical water as the extraction media and analysing the results, it has to be considered that the properties of water are changed by changing the operating parameters. By increasing the temperature, the polarity of water decreases while the ionization constant and reactivity increase. The extraction yield is therefore influenced by the two competing effects, i.e., solubility in media and the degradation rate. At 150 and 200 °C, the mass transfer rate was higher when the solvent–solid ratio was higher due to the higher concentration difference between the actual and equilibrium concentration of solutes in the media, while the degradation rate was low. However, at higher temperature (250 °C), the ionization constant of water is higher, thus a higher yield was obtained for a lower solvent–solid ratio (10 mL/g) where the degradation rate was lower due to a lower concentration of ions in the media. By comparing the results of the present study with data published by Živković et al. [3], it can be observed that ultrasound-assisted extraction of chestnut bark with 50% ethanol gave significantly lower extraction yields (7.84% for new and 3.40% (*w/w*) for old chestnut bark) than that obtained by subcritical water extraction. Furthermore, Vázquez et al. [14] used different media for the conventional extraction of chestnut shells, and water was shown as the most efficient extraction solvent (extraction yield of 12.2% at 90 °C), and when Na_2_SO_3_ was added into water it gave even higher extraction yield of 25.62%. It can be concluded that subcritical water extraction of chestnut bark gave significantly higher extraction yield (44.9% at 150 °C and 10 min) than the extraction of chestnut shell with water.

The second-order polynomial model was proposed for obtaining predicted extraction yield (Y_1_):

Y_1_ = 21.46 − 3.41X_1_ − 5.01X_2_ + 1.94X_3_ + 2.63 X_1_X_2_ − 2.52X_1_X_3_ + 1.35 X_2_X_3_ + 7.08X_1_^2^ + 4.71X_2_^2^ − 1.90X_3_^2^ (1)

where X_1_, X_2_, and X_3_ are, in terms of coded factors of the test variables, temperature, time, and solvent-solid ratio, respectively.

All regression coefficients (intercept, linear, quadratic and interaction terms) are significant and have an influence on extraction yield. The values of regression coefficients and analysis of variance (ANOVA) of the experimental results obtained for extraction yield are presented in Table 2. The F-value of 64.84 and *p*-value lower than 0.0001 of the model imply that the model is significant. The lack of fit is not significant, with an *F*-value of 0.43 and *p*-value of 0.7440. The *R*^2^ value of 0.9881 shows the good quality of the model. The predicted *R*^2^ value (0.9729) is in good agreement with the adjusted one (0.9399). The adequate precision of 33.461 is greater than 4, which makes the signal to noise ratio adequate. Therefore, the analysis of the model confirmed that the model fits with the experimental results of extraction yield.

**Figure 1:**
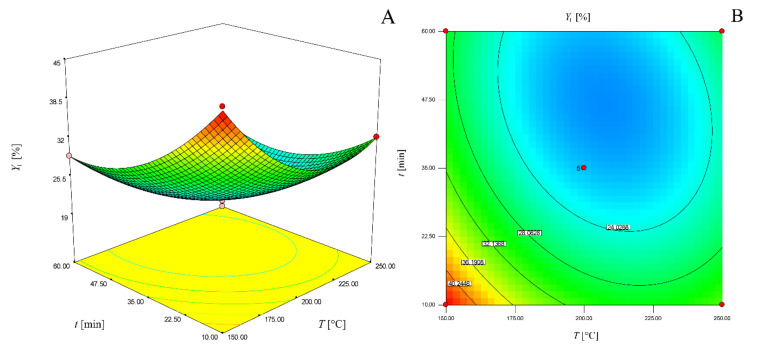
Three-dimensional response surface (**a**) and two-dimensional contour plot (**b**) of the model for determination of chestnut bark extraction yield for solvent-solid ratio of 20 mL/g.

Figure 1 represents the three-dimensional response surface (a) and two-dimensional contour plot (b) of the model for solvent-solid ratio of 20 mL/g which can be used for the determination of chestnut bark extract yields.

### 2.2. Total Phenols, Total Tannins and Antioxidant Activity

Table 3 represents the obtained content of phenols, tannins and antioxidant activity for the same mentioned experiments determined by the Box-Behnken method. The highest total phenol content of 85.23 mg/g of chestnut bark was obtained at 150 °C, 35 min and 30 mL/g. Accordingly, as tannins are the main part of the chestnut bark structure, the highest yield of total tannins of 98 mg/g of chestnut bark was obtained at the same conditions. The total phenol and tannin contents decrease with the increase in the temperature, extraction time and solvent-solid ratio. Opposite to this, antioxidant activity generally increases with the increasing temperature and extraction time. The comparison of the results with the published data shows that dry extract of chestnut bark obtained by ultrasound assisted extraction with 50% ethanol [3] contained lower amounts of total phenols (3% for new and 1.7% for old chestnut bark) than extracts obtained by hydrothermal extraction (the highest concentration of total phenols in extract of 327.0 mg/g of extract (32.7%) was obtained at 250 °C, 35 min and 30 mL/g ).

The proposed quadratic models for determination of total phenol (Y_2_) content, total tannin (Y_3_) content and antioxidant activity (Y_4_) are as follows:

Y_2_ = 57.50 − 6.25X_1_ − 5.58X_2_ + 12.31X_3_ + 10.68X_1_^2^ (2)

Y_3_ = 69.63 − 5.89X_1_ − 3.54X_2_ + 15.24X_3_ + 9.38X_1_^2^ − 3.37X_3_^2^ (3)

Y_4_ = 87.56 + 5.20X_1_ − 0.96X_3_ +2.56 X_1_ X_3_ −1.38X_2_ X_3_ − 4.11X_1_^2^ +1.62X_2_^2^− 1.39X_3_^2^ (4)

The regression coefficients, such as X_1_ X_2_, X_1_ X_3_, X_2_ X_3_, X_2_^2^ and X_3_^2^, are not significant and have no influence on the total phenol content. The regression coefficients which are insignificant for the determination of total tannins are all interaction terms and X_2_^2^. In the case of the antioxidant activity model, X_2_ and X_1_ X_2_ are insignificant. The values of regression coefficients and the analysis of variance (ANOVA) of the experimental results obtained for total phenol content, total tannin content and antioxidant activity are presented in Table 4. The models for determination of total phenols, total tannins and antioxidant activity due to obtained *F*-values (31.11, 43.73 and 24.39, respectively) and *p*-values (<0.0001) are significant. The lack of fit is not significant for each response (*p* > 0.05). The signal to noise ratio is adequate for each response, due to the value of adequate precision being higher than 4. *R*^2^ values of 0.9121, 0.9521 and 0.9499 of the models for total phenols, total tannins and antioxidant activity determination, respectively, are satisfactory. 

**Figure 2:**
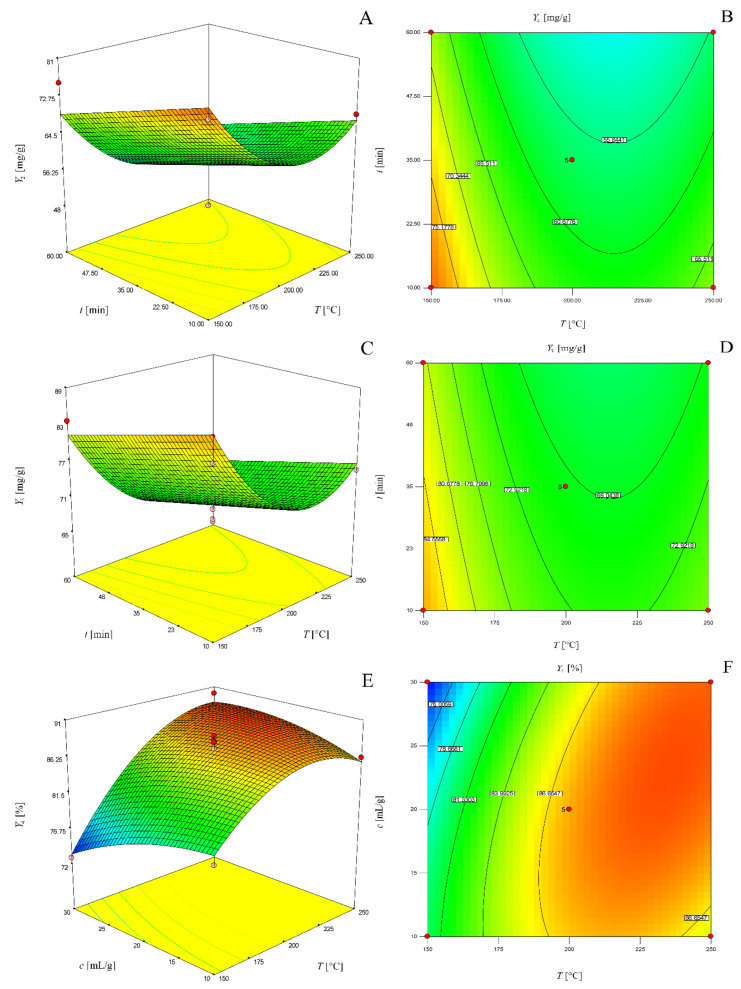
Three-dimensional response surface (**a**) and two-dimensional contour plot (**b**) of the model for determination of total phenol content for solvent-solid ratio of 20 mL/g; three-dimensional response surface (**c**) and two-dimensional contour plot (**d**) of the model for determination of total tannin content for solvent-solid ratio of 20 mL/g; three-dimensional response surface (**e**) and two-dimensional contour plot (**f**) of the model for determination of antioxidant activity for 35 min.

The graphical representation of these models as three-dimensional response surfaces and two-dimensional contour plots are presented in Figure 2.

### 2.3. Ellagic Acid, Gallic Acid and Ellagitannins

Table 5 represents the obtained yields of ellagic acid, gallic acid, vescalagin, castalagin and 1-*o*-galloyl-castalagin for the same mentioned experiments determined by Box-Behnken method. It can be noticed that the yield of ellagic acid increases with the increase in temperature, but decreases as the time gets longer. At 150 °C, the yield of gallic acid increases when increasing the extraction time, while at 200 °C, it decreases as the time increases. Although chestnut bark contains mainly ellagitannins, it also might contain gallotannins, which contain gallic acid [9]. Furthermore, ellagic acid, which is the main product of ellagitannins [28], is formed by the condensation of two gallic acid molecules [29]. The experimental results show that the yield of ellagitannins decreases when the temperature increases, while at a temperature of 150 °C, it increases from 10 to 35 min and then decreases. Ellagitannins are not stable and are hydrolyzed at high temperatures under subcritical conditions into ellagic acid [1]. In our previous work, where the hydrothermal hydrolysis of chestnut tannin extract [1] was studied, it was observed that the content of ellagitannins was the highest when sweet chestnut tannins were treated by subcritical water at 120 °C. Furthermore, as in our previous work [1], the highest concentration of ellagic acid in extracts was obtained at 250 °C; however, longer treatment time was needed in the present work for the extraction and simultaneous hydrolysis of tannins from chestnut bark. Based on the results, it can be concluded that the degradation rate of ellagic acid is probably slower than the rate of its production by the hydrolysis of ellagitannins. Vescalin and castalin are present in trace amounts at low temperature of 150 °C. Gallic acid and ellagitannins were no longer present in the samples above 200 °C. Furthermore, the solvent–solid ratio of 30 mL/g gives higher yields of almost all these compounds than a ratio of 10 mL/g. 

Comandini et al. [9] and Chiarini et al [7] obtained higher contents of ellagitannins and gallic acid in extracts when chestnut bark was treated with methanol, but the ellagic acid content in the extracts was lower than that obtained by subcritical water extraction (Table 6). 

Only ellagic acid was used for the optimization process, due to its presence in all bark extracts. The quadratic model for the determination of ellagic acid yield (Y_5_) was proposed, as follows:

Y_5_ = 11.68 − 1.18X_2_ + 3.07X_3_ − 0.85X_1_X_2_ + 0.87X_2_X_3_ − 1.45X_2_^2^ (5)

The values of regression coefficients and the analysis of variance (ANOVA) of the experimental results of ellagic acid yield are presented in Table 7. The regression coefficients X_1,_ X_1_X_3_, X_1_^2^ and X_3_^2^ are insignificant and are removed from the equation because they have no influence on the yield. The model’s *F*-value of 35.97 and *p*-value lower than 0.0001 imply that model is significant. The lack of fit’s *F*-value of 0.33 and *p*-value of 0.9042 show that the lack of fit is insignificant. The *R*^2^ value of 0.9424 shows the good quality of the model and reasonable agreement between the predicted *R*^2^ value of 0.8711 and adjusted *R*^2^ value of 0.9162. The adequate precision is good due to the value of 21.613 being greater than 4. Therefore, from the analysis of the proposed model of ellagic acid yield, it can be concluded that it can be used to navigate the design space.

**Figure 3:**
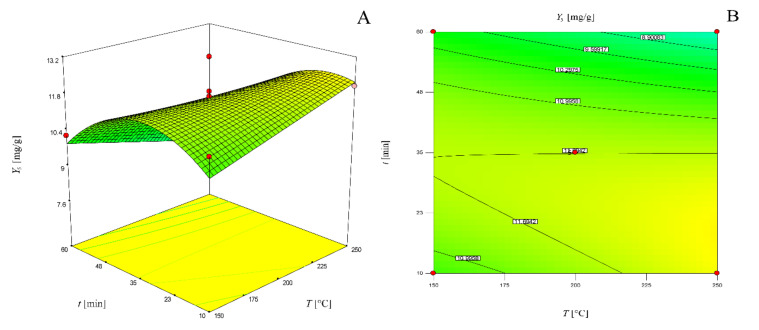
Three-dimensional response surface (**a**) and two-dimensional contour plot (**b**) of the model for determination of ellagic acid yield for solvent-solid ratio of 20 mL/g.

The graphical representations of ellagic acid yield dependent on time and temperature for a solvent–solid ratio of 20 mL/g as three-dimensional response surface and two-dimensional contour plots are presented in Figure 3.

### 2.4. Sugars and Their Derivatives

The yields of sugars (glucose, fructose, maltose, and arabinose) obtained by the performed experiments determined by the Box–Behnken method are presented in Table 8. It is obvious that sugar yield decreases with extraction time and temperature. Glucose and fructose yields were higher if a solvent–solid ratio of 10 mL/g was used, while maltose yield was higher at conditions where solvent-solid ratio was 30 mL/g. Furthermore, no sugars were present in extracts obtained at 250 °C. Arabinose was present only in bark extracts obtained at 150 and at 200 °C and 10 min, but in much lower yield than other sugars. From the results, it can be concluded that starch is a part of the chestnut bark, because its degradation leads to the formation of maltose [30]. Furthermore, glucose and fructose are probably cellulose degradation products [31], while arabinose is obtained from hemicellulose degradation [32]. Glucose and arabinose were also detected in chestnut shell extracts obtained by hydrothermal extraction [33]. Similarly to the present work, arabinose content increased with increasing the temperature up to 200 °C, then decreased, and at higher temperatures (above 210 °C), it was not present in the extract anymore, while glucose content increased with increasing the temperature up to 215 °C and then decreased [33].

The quadratic models for the determination of glucose (*Y*_6_), fructose (*Y*_7_) and maltose (*Y*_8_) yields could be improved if the responses (glucose, fructose and maltose yield) are square root transformed:

Sqrt(Y_6’_) = 3.35 − 1.57X_1_ − 0.30X_2_ − 0.11X_3_ + 0.21X_1_X_2_ − 1.78X_1_^2^ (6)

Sqrt(Y_7’_) = 3.41 − 1.78X_1_ − 0.25X_2_ − 1.63X_1_^2^ (7)

Sqrt(Y_8’_) = 1.98 − 1.79X_1_ − 0.35X_2_ + 0.077X_3_ + 0.33X_1_X_2_ − 0.12X_1_X_3_ − 0.19X_1_^2^ (8)

Analysis of variance (ANOVA) of the experimental results obtained for glucose, fructose and maltose yields is presented in Table 9. The arabinose yield was not optimized due to insufficient presence in the samples. The regression coefficients such as X_1_ X_3_, X_2_ X_3_, X_2_^2^ and X_3_^2^ are not significant and have no influence on glucose yield, while in the model for maltose yield, X_2_ X_3_, X_2_^2^ and X_3_^2^ are removed from the equation because they are insignificant. The regression coefficients which are insignificant in model for fructose yield are all interaction terms (X_1_ X_2,_ X_1_ X_3_, X_2_ X_3_), X_3_, X_2_^2^ and X_3_^2^. The models for the determination of glucose, fructose and maltose yield due to the obtained *F*-values (350.71, 212.08, 338.9, respectively) and *p*-values (<0.0001) are significant. The lack of fit is not significant for each response (*p* > 0.05). The adequate precision of each response indicates an adequate signal (ratio > 4). The *R*^2^ values show the good quality of the model. The predicted *R*^2^ values and adjusted *R*^2^ values are in reasonable agreement. Models can be used to navigate the design space.

**Figure 4:**
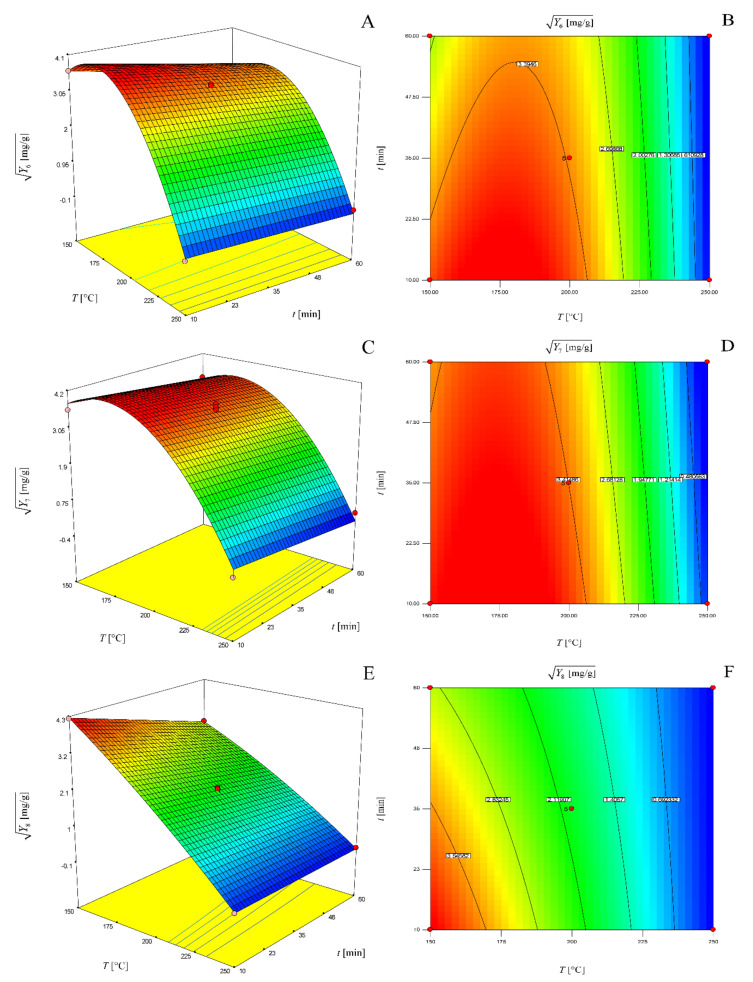
Three-dimensional response surface (**a**) and two-dimensional contour plot (**b**) of the model for determination of glucose yield for a solvent–solid ratio of 20 mL/g; three-dimensional response surface (**c**) and two-dimensional contour plot (**d**) of the model for determination of fructose yield for a solvent–solid ratio of 20 mL/g; three-dimensional response surface (**e**) and two-dimensional contour plot (**f**) of the model for determination of maltose yield for a solvent–solid ratio of 20 mL/g.

The graphical representations of these models as three-dimensional response surfaces and two-dimensional contour plots are presented in Figure 4.

The yields of sugar derivatives (levulinic acid, 5-HMF and furfural) obtained by performed experiments determined by the Box–Behnken method are presented in Table 10. Obviously, as sugar yield decreases, 5-HMF, furfural and levulinic acid yields increase, due to the hydrothermal degradation of sugars at higher temperatures and times. 5-HMF and furfural yields increase with increasing temperature. Furthermore, at lower temperatures (150 and 200 °C), the yields of 5-HMF and furfural increase with time, while at a temperature of 250 °C, yields decrease as the time increases. The higher yields of 5-HMF and furfural were generally obtained by using a solvent–solid ratio of 10 rather than 30 mL/g. Levulinic acid appears at 200 °C and 35 min and its yield increases with temperature and time. The solvent–solid ratio has no significant influence on levulinic acid yield. Gullon et al. [33] showed that furfural and 5-HMF content in chestnut shell extracts obtained by hydrothermal extraction increased with increasing the temperature, which was also shown in the present work. Further, the furfural content was higher than 5-HMF content [33], which is also obtained in the present work.

The quadratic models for determination of 5-HMF (Y_9_) and furfural (Y_10_) yields could be improved if the responses (5-HMF and furfural yield) are natural logarithmic-transformed:

Ln(Y_9_) = 2.10 + 1.32X_1_ + 0.34X_2_ − 0.12X_3_ − 0.64X_1_X_2_ + 0.35X_1_X_3_ − 1.55X_1_^2^ − 0.25X_2_^2^ (9)

Ln(Y_10_) = 2.82 + 1.32X_1_ + 0.28X_2_ − 0.12 X_3_ − 0.87X_1_X_2_ + 0.40X_1_X_3_ − 2.47X_1_^2^ − 0.21X_2_^2^ (10)

The analysis of variance (ANOVA) of the experimental results obtained for 5-HMF and furfural yields is presented in Table 11. The regression coefficients such as X_2_ X_3_ and X_3_^2^ are not significant for both models and are removed from the equations because they have no influence on the determination of 5-HMF and furfural yields. These models are significant due to the obtained *p*-values being lower than 0.0001. The lack of fit is not significant due to p-values higher than 0.05. The adequate precision of each response indicates an adequate signal (ratio > 4). The *R*^2^ values of 0.9927 and 0.9954 for 5-HMF and furfural model, respectively, show the excellent quality of the models. Predicted *R*^2^ values are in reasonable agreement with the adjusted *R*^2^ values. Therefore, the models fit to the experimental data.

**Figure 5:**
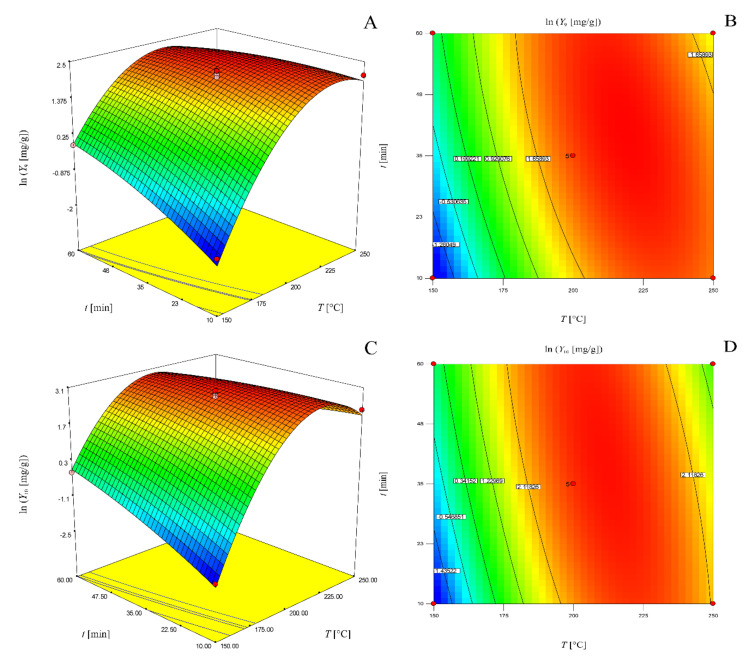
Three-dimensional response surface (**a**) and two-dimensional contour plot (**b**) of the model for determination of 5-HMF yield for a solvent–solid ratio of 20 mL/g; three-dimensional response surface (**c**) and two-dimensional contour plot (**d**) of the model for determination of furfural yield for a solvent–solid ratio of 20 mL/g.

The graphical representations of these models as three-dimensional response surfaces and two-dimensional contour plots are presented in Figure 5.

### 2.5. Optimal Conditions

Due to positive health effects of ellagic acid, which was present in all bark extracts, the aim was to determine the optimal conditions of subcritical water extraction which maximize the yield of ellagic acid and at the same time give a satisfactory yield of total phenols and tannins. Different solutions were offered by the program. Although the highest yield of ellagic acid was obtained for conditions of 250 °C, 29 min and 30 mL/g (ellagic acid—14.9 mg/g of bark, total phenols—77.3 mg/g of bark, total tannins—86.9 mg/g of bark), a practically negligibly lower yield of ellagic acid (14.8 mg/g of bark) was obtained at conditions of 150 °C, 35 min and 30 mL/g, but the yields of total phenols and total tannins were higher (86.8 and 96.8 mg/g of bark). Furthermore, it is more economical to use lower temperatures due to lower energy consumption. Therefore, the optimal conditions were chosen to be 150 °C, 36 min and 30 mL/g. The yields of ellagic acid, total phenols and total tannins obtained by experiment performed at determined optimal conditions were 14.2, 85.2 and 98.3 mg/g of bark, respectively (41.89 mg/g, 251.33 mg/g and 289.97 mg/g of extract, respectively), which is in reasonable agreement with the predicted values and thus suggests that the models are valid.

## 3. Materials and Methods

### 3.1. Materials

The sweet chestnut bark was obtained from a local company Tannin Sevica (Slovenia). Gallic, ellagic and levulinic acids, sugars, 5-HMF, furfural, Na_2_CO_3_ and phenol were obtained from Sigma-Aldrich (Steinheim, Germany). Folin-Dennis and Folin-Ciocalteu reagents and sulfuric acid (95–97%) were obtained from Merck (Darmstadt, Germany). All other chemicals used for HPLC were of analytical grade.

### 3.2. Subcritical Water Extraction

Subcritical water extraction was performed in 75 mL batch reactor (series 4740 stainless steel, Parr instruments, Moline, IL, USA) at temperatures from 150 to 250 °C and at times from 10 to 60 min. Different water-bark (solvent-solid) ratios were prepared (10, 20, and 30 mL/g). The mixture of the bark and water was poured into the reactor. The reactor was heated by electrical wire. The heating rate was around 23 °C per minute, so the temperature of 250 °C was reached in approximately 11 min. Nitrogen was used to remove present oxygen from the reactor and to control the pressure, that was adjusted to 45 bar in all experiments. Nitrogen was used to remove present oxygen from the reactor and to control the pressure. The mixture was mixed at 600 rpm. The time was measured from the moment when the desired temperature was reached. After the extraction, the reactor was exposed to rapid cooling. The mixture was cooled within 6 minutes. The reactor content was filtrated by vacuum filtration and the solvent from the extract was evaporated using a rotary evaporator at low pressure and at 40 °C. Further, extract was analyzed by HPLC and UV spectrophotometer. The yield of the obtained bark extracts was calculated using Equation (11):

(11) $$Y\;\left(\%\right)=\frac{m_{extract}}{m_{raw\;material}}\cdot 100\;\%$$

### 3.3. Total Phenols

The Folin–Ciocalteu method [34] was used for determination of total phenol content in extracts [35]. In this process, 2.5 mL of Folin–Ciocalteu reagent (diluted with water 1:10) and 2 mL of Na_2_CO_3_ solution (75 g/L) were added to 0.5 mL of bark extract. The sample was heated in a water bath at a temperature of 50 °C for 5 min and then it was cooled at room temperature for 30 min. The absorbance was measured at a wavelength of 760 nm by an UV-VIS spectrophotometer (Cary 50, Varian, Palo Alto, CA, USA). The control sample was prepared in the same way using water instead of bark extract. The calibration curve was prepared using gallic acid standard and the total phenol content was expressed in mg GA/g of bark. 

### 3.4. Total Tannins

The Folin–Dennis method [34] was used for the determination of total tannin content in extracts [36]. To 1 mL of extract, 2.5 mL of Folin–Dennis reagent (diluted with water 1:10) and 2 mL of Na_2_CO_3_ solution (75 g/L) were added. The samples were kept at room temperature for a 30 min, after which the absorbance of the samples was measured at 760 nm by an UV-VIS spectrophotometer. The deionized water was used for the preparation of the control sample instead of the extract. The quantification was done by preparing a calibration curve with tannic acid and results were expressed in mg TA/g of bark.

### 3.5. Antioxidant Activity

Antioxidant activity of water-soluble products obtained by hydrothermal treatment of sweet chestnut bark was determined by DPPH method [37]. In this process, 77 μL of the extract solution (concentration of 1 mg/mL) was mixed with 3 mL of DPPH solution and incubated in a dark room for 15 min. The absorbance of the sample was measured at 515 nm using an UV-VIS spectrophotometer. The control sample was prepared in the same way using methanol instead of bark extract, but its absorbance was measured immediately. Antioxidant activity was calculated using Equation (12) and expressed in %:

(12) $$\%\;DPPH\;activity=\frac{A_{c}^{0}-A_{s}^{15}}{A_{c}^{0}}\;\cdot 100\;\%$$

where $A_{c}^{0}$ is the absorbance of the control sample, while $A_{s}^{15}$ is the absorbance of the extract samples.

### 3.6. HPLC Analysis

The analysis of extracts was performed by an Agilent 1100 Series HPLC system (Waldbronn, Germany) with C18 (4.0 × 250 mm, 5 μm particle size) column. The method used was taken from the literature [9] with minor changes. The mobile phases were water-formic acid (99.5:0.5) (solvent A) and acetonitrile (solvent B). The gradient was as follows: 0 to 2 min 5% B, from 2 to 10 min 5–20% B, from 10 to 15 min 20–30% B, from 15 to 20 min 30–35% B, from 20 to 60 min 35–80% B, from 60 to 65 min 80–85%, from 65 to 70 min 85–5% B. The flow rate was 0.89 mL/min. The column temperature was 25 °C. The injector volume of samples was 20 μl. The wavelengths were set to 254 and 280 nm. The quantification of compounds was done by standard curves of gallic acid measured at 280 nm (*r*^2^ = 0.9991) and ellagic acid measured at 254 nm (*r*^2^ = 0.9992). The obtained ellagitannins were identified by comparing the retention times and UV spectrum to the literature data [9]. The ellagitannins (vescalin, castalin, vescalagin, castalagin and 1-*o*-galloyl castalagin) were quantified using the calibration curve of ellagic acid with applied correlation factors: for vescalin and castalin (6322/302), for vescalagin and castalagin (934/302), for 1-*o*-galloyl castalagin (1086/302) [9].

The sugars and their derivatives found in extracts were analyzed by the HPLC method described in our previous work [31]. The extracts were analyzed by a Shimadzu Nexera HPLC system with RI detector (for sugar detection) and UV detector (for detection of sugar derivatives). The column used was Rezex RHMMonosaccharide H+ (300 × 7.8 mm) at 80 °C. The method was isocratic, and water was used as mobile phase with the flow rate of 0.6 mL/min. The quantification of obtained products was performed using calibration curves of standards.

### 3.7. Statistical Analysis

The analyses of extracts were repeated three times. Each data point represents the average of three measurements and the relative standard deviation between measurements was 1%.

### 3.8. Optimization of Reaction Parameters

The response surface methodology, i.e., Box–Behnken design (BBD), was chosen for the optimization of reaction parameters of subcritical water extraction of chestnut bark and the software used was Design Expert 7.0.0 Trial version (Stat-Ease, Inc., Minneapolis, MN, USA). The three variables were optimized: temperature (X_1_, ˚C) in range from 150 ˚C to 250 ˚C, time (X_2_, min) in range from 10 to 60 min and a solvent–solid ratio (X_3_, mL/g) in range from 10 to 30 mL/g. The whole design consisted of 17 experimental points. Five replicates of center points were used for determination of a pure error sum of squares. 

Experimental data were fitted with second order response surface model with the following Equation (13):

(13) $$Y=\beta_{0}+\overset{k}{\underset{i=1}{\sum }}\beta_{i}X_{i}+\overset{k}{\underset{i=1}{\sum }}\beta_{ii}X_{i}^{2}+\overset{k-1}{\underset{\begin{array}{c} i=1\\ i<j \end{array}}{\sum }}\overset{k}{\underset{j=2}{\sum }}\beta_{ij}X_{i}X_{j}$$

where *Y* is the investigated response (extraction yield or amount of extracted components), *β*_0_, *β*_i_, *β*_ii_, *β*_ij_ are constant coefficients of the intercept, linear, quadratic, and interaction terms, respectively; *X*ì and *X*j are coded independent variables.

The Box–Behnken method proposed experiments which should be done in a certain range of factor variables and then suggested the mathematical model based on results obtained by the experiments. Finally, the optimal process conditions to obtain the product with high level of total phenols, tannins and ellagic acid were calculated and were compared to the results obtained by the experiment.

## 4. Conclusions

The results of the present study show that besides ellagic acid as the main compound of the bark extract, high amounts of gallic acid and ellagitannins are also present in the chestnut bark extract. The health benefits of these compounds have been intensively studied before [4,10,11,38]. Further compounds present in the chestnut bark extract include sugars, mainly glucose and fructose, and in also lower amounts arabinose and maltose. The study shows that higher contents of ellagitannins, gallic acid, sugars, total tannins and total phenols are obtained at lower extraction temperature and time, due to the reduced stability of the molecules in harsh conditions. On the other hand, it can also be noticed that sugar derivatives (5-HMF, furfural, levulinic acid) are formed in higher amounts at higher temperatures and longer times. Although ellagic acid yield was the highest at conditions of 250 °C, 29 min and 30 mL/g (14.9 mg/g of bark), a practically negligibly lower amount was predicted at conditions of 150 °C, 35 min and 30 mL/g (14.8 mg/g of bark), which were also chosen as the optimal process conditions. The experimentally determined yields of ellagic acid, total tannins and total phenols obtained by extraction at optimal conditions were in agreement with the predicted values and were 14.2, 98.3 and 85.2 mg/g of bark, respectively, which shows the validity of the models. The high content of ellagic acid at 250 °C shows the high stability of the molecule, but from the economic point of view and by taking into account the thermostability of compounds, the extraction at a lower temperature is preferable.

## Figures and Tables

**Table 1:** Proposed experiments by the Box–Behnken method and the predicted and experimental values of extraction yield obtained by subcritical water extraction of chestnut bark.

Run	Temp. (X_1_), °C	Time (X_2_), min	Solvent-Solid Ratio (X_3_), mL/g	Extraction Yield, %	
				Experimental	Predicted
**1**	150 (−1)	10 (−1)	20 (0)	44.9	44.3
**2**	250 (1)	10 (−1)	20 (0)	32.2	32.2
**3**	150 (−1)	60 (1)	20 (0)	29.0	29.0
**4**	250 (1)	60 (1)	20 (0)	26.9	27.5
**5**	150 (−1)	35 (0)	10 (−1)	25.6	25.6
**6**	250 (1)	35 (0)	10 (−1)	24.4	23.8
**7**	150 (−1)	35 (0)	30 (1)	33.9	34.5
**8**	250 (1)	35 (0)	30 (1)	22.6	22.6
**9**	200 (0)	10 (−1)	10 (−1)	28.1	28.7
**10**	200 (0)	60 (1)	10 (−1)	15.9	16.0
**11**	200 (0)	10 (−1)	30 (1)	29.9	29.9
**12**	200 (0)	60 (1)	30 (1)	23.1	22.6
**13**	200 (0)	35 (0)	20 (0)	20.3	21.5
**14**	200 (0)	35 (0)	20 (0)	23.3	21.5
**15**	200 (0)	35 (0)	20 (0)	21.3	21.5
**16**	200 (0)	35 (0)	20 (0)	22.1	21.5
**17**	200 (0)	35 (0)	20 (0)	20.3	21.5

**Table 2:** The values of regression coefficients and analysis of the model for extraction yield.

Parameter	Coefficient Estimate	Standard Error	Sum of Squares	Degrees of Freedom	Mean Square	*F*-Value	*p*-Value Probability > *F*
**Model**			710.81	9	78.98	64.84	<0.0001
**Intercept**	21.46	0.49		1			
**X_1_**	−3.41	0.39	93.02	1	93.02	76.37	<0.0001
**X_2_**	−5.01	0.39	200.70	1	200.70	164.77	<0.0001
**X_3_**	1.94	0.39	29.99	1	29.99	24.62	0.0016
**X_1_ X_2_**	2.63	0.55	27.72	1	27.72	22.76	0.0020
**X_1_ X_3_**	−2.52	0.55	25.35	1	25.35	20.81	0.0026
**X_2_ X_3_**	1.35	0.55	7.24	1	7.24	5.94	0.0449
**X_1_^2^**	7.08	0.54	211.03	1	211.03	173.25	<0.0001
**X_2_^2^**	4.71	0.54	93.49	1	93.49	76.75	<0.0001
**X_3_^2^**	−1.90	0.54	15.25	1	15.25	12.52	0.0095
**Residual**			8.53	7	1.22		
**Lack of fit**			2.07	3	0.69	0.43	0.7440
**Pure error**			6.45	4	1.61		
***R*^2^**	0.9881		**Adj *R*^2^**	0.9729			
**C.V. %**	4.23		**Pred *R*^2^**	0.9399			
**PRESS**	43.25		**Adeq Precision**	33.461			

**Table 3:** Proposed experiments by the Box–Behnken method and the predicted and experimental values of total phenol content, total tannin content and antioxidant activity of extracts obtained by subcritical water extraction of chestnut bark.

Run	Temp. (X_1_), °C	Time (X_2_), min	Solvent-Solid Ratio (X_3_), mL/g	Total Phenol Content, mg/g of Bark		Total Tannin Content, mg/g of Bark		Antioxidant Activity, %	
				Experimental	Predicted	Experimental	Predicted	Experimental	Predicted
**1**	150 (−1)	10 (−1)	20 (0)	77.7	80.0	84.4	88.4	80.5	79.9
**2**	250 (1)	10 (−1)	20 (0)	68.8	67.5	75.5	76.7	90.6	90.3
**3**	150 (−1)	60 (1)	20 (0)	75.7	68.9	83.6	81.4	81.0	79.9
**4**	250 (1)	60 (1)	20 (0)	57.8	56.4	73.9	69.6	88.2	90.3
**5**	150 (−1)	35 (0)	10 (−1)	59.1	62.1	66.6	66.3	79.2	80.4
**6**	250 (1)	35 (0)	10 (−1)	47.3	49.6	53.3	54.5	86.2	85.7
**7**	150 (−1)	35 (0)	30 (1)	85.2	86.7	98.3	96.8	72.8	73.3
**8**	250 (1)	35 (0)	30 (1)	73.9	74.2	83.0	85.0	90.0	88.9
**9**	200 (0)	10 (−1)	10 (−1)	53.7	50.8	60.3	54.6	88.1	87.4
**10**	200 (0)	60 (1)	10 (−1)	38.3	39.6	42.8	47.5	90.0	90.1
**11**	200 (0)	10 (−1)	30 (1)	76.9	75.4	86.0	85.0	88.3	88.2
**12**	200 (0)	60 (1)	30 (1)	60.8	64.2	77.5	78.0	84.7	85.5
**13**	200 (0)	35 (0)	20 (0)	59.6	57.5	66.7	69.6	85.3	87.6
**14**	200 (0)	35 (0)	20 (0)	48.1	57.5	72.6	69.6	87.3	87.6
**15**	200 (0)	35 (0)	20 (0)	61.9	57.5	67.1	69.6	88.9	87.6
**16**	200 (0)	35 (0)	20 (0)	60.2	57.5	68.9	69.6	88.3	87.6
**17**	200 (0)	35 (0)	20 (0)	58.1	57.5	71.5	69.6	88.1	87.6

**Table 4:** The values of regression coefficients and analysis of the models for total phenols, total tannins and antioxidant activity.

TOTAL PHENOLS							
Parameter	Coefficient Estimate	Standard Error	Sum of Squares	Degrees of Freedom	Mean Square	*F*-Value	*p*-Value Probability > *F*
**Model**			2256.04	4	78.98	31.11	<0.0001
**Intercept**	57.50	1.42		1			
**X_1_**	−6.25	1.51	312.50	1	312.50	17.24	0.0013
**X_2_**	−5.58	1.51	248.98	1	248.98	13.73	0.0030
**X_3_**	12.31	1.51	1211.55	1	1211.55	66.83	<0.0001
**X_1_^2^**	10.68	2.07	483.01	1	483.01	26.64	0.0002
**Residual**			217.54	12	18.13		
**Lack of fit**			96.80	8	12.10	0.40	0.8738
**Pure error**			120.74	4	30.19		
***R*^2^**	0.9121		**Adj R^2^**	0.8827			
**C.V. %**	6.81		**Pred R^2^**	0.8392			
**PRESS**	397.87		**Adeq Precision**	20.407			
**TOTAL TANNINS**							
**Parameter**	**Coefficient Estimate**	**Standard Error**	**Sum of Squares**	**Degrees of Freedom**	**Mean Square**	***F*-value**	***p*-value** **Probability > *F***
**Model**			2641.11	5	528.22	43.73	<0.0001
**Intercept**	69.63	1.38		1			
**X_1_**	−5.89	1.23	277.18	1	277.18	22.95	0.0006
**X_2_**	−3.54	1.23	100.32	1	100.32	8.31	0.0149
**X_3_**	15.24	1.23	1858.06	1	1858.06	153.82	<0.0001
**X_1_^2^**	9.38	1.69	371.28	1	371.28	30.74	0.0002
**X_3_^2^**	−3.37	1.69	47.81	1	47.81	3.96	0.0721
**Residual**			132.88	11	12.08		
**Lack of fit**			105.72	7	15.10	2.22	0.2294
**Pure error**			27.16	4	6.79		
***R*^2^**	0.9521		**Adj *R*^2^**	0.9303			
**C.V. %**	4.80		**Pred *R*^2^**	0.8674			
**PRESS**	367.89		**Adeq Precision**	23.869			
**ANTIOXIDANT ACTIVITY**							
**Parameter**	**Coefficient Estimate**	**Standard Error**	**Sum of Squares**	**Degrees of Freedom**	**Mean Square**	***F*-value**	***p*-value** **Probability > *F***
**Model**			346.89	7	49.56	24.39	<0.0001
**Intercept**	87.56	0.64		1			
**X_1_**	5.20	0.50	216.32	1	216.32	106.48	<0.0001
**X_3_**	−0.96	0.50	7.32	1	7.32	3.60	0.0902
**X_1_ X_3_**	2.56	0.71	26.16	1	26.16	12.88	0.0059
**X_2_ X_3_**	−1.38	0.71	7.62	1	7.62	3.75	0.0848
**X_1_^2^**	−4.11	0.69	71.19	1	71.19	35.04	0.0002
**X_2_^2^**	1.62	0.69	11.06	1	11.06	5.44	0.0445
**X_3_^2^**	−1.39	0.69	8.19	1	8.19	4.03	0.0756
**Residual**			18.28	9	2.03		
**Lack of fit**			10.37	5	2.07	1.05	0.4959
**Pure error**			7.92	4	1.98		
***R*^2^**	0.9499		**Adj *R*^2^**	0.9110			
**C.V. %**	1.66		**Pred *R*^2^**	0.7557			
**PRESS**	89.20		**Adeq Precision**	17.313			

**Table 5:** Proposed experiments by the Box–Behnken method and the predicted and experimental values of yield of ellagic and gallic acid and ellagitannins obtained by subcritical water extraction of chestnut bark.

Run	Temp. (X_1_), °C	Time (X_2_), min	Solvent-Solid Ratio (X_3_), mL/g	Ellagic Acid, mg/g of Bark		Gallic Acid, mg/g of Bark	Vescalagin, mg/g of Bark	Castalagin, mg/g of Bark	1-*o*-galloyl-Castalagin, mg/g of Bark
				Experimental	Predicted	Experimental	Experimental	Experimental	Experimental
1	150 (−1)	10 (−1)	20 (0)	11.3	10.6	2.8	1.1	5.3	1.6
2	250 (1)	10 (−1)	20 (0)	12.1	12.3	0	0	0	0
3	150 (−1)	60 (1)	20 (0)	10.2	9.9	3.3	traces	2.3	0.4
4	250 (1)	60 (1)	20 (0)	7.6	8.2	0	0	0	0
5	150 (−1)	35 (0)	10 (−1)	9.3	8.6	3.2	traces	3.4	0.3
6	250 (1)	35 (0)	10 (−1)	8.5	8.6	0	0	0	0
7	150 (−1)	35 (0)	30 (1)	14.2	14.8	3.5	3.1	5.3	0.4
8	250 (1)	35 (0)	30 (1)	14.8	14.8	0	0	0	0
9	200 (0)	10 (−1)	10 (−1)	8.7	9.2	2.8	traces	0.09	0.01
10	200 (0)	60 (1)	10 (−1)	5.0	5.1	0.6	0	0	0
11	200 (0)	10 (−1)	30 (1)	13.6	13.6	3.7	traces	0.2	trace
12	200 (0)	60 (1)	30 (1)	13.4	13.0	Traces	0	0	0
13	200 (0)	35 (0)	20 (0)	11.7	11.7	1.2	0	0	0
14	200 (0)	35 (0)	20 (0)	11.9	11.7	1.2	0	0	0
15	200 (0)	35 (0)	20 (0)	10.9	11.7	1.2	0	0	0
16	200 (0)	35 (0)	20 (0)	13.2	11.7	1.2	0	0	0
17	200 (0)	35 (0)	20 (0)	10.7	11.7	0.9	0	0	0

**Table 6:** Concentration of ellagitannins, gallic acid and ellagic acid in chestnut bark extracts obtained by different methods.

Compounds	Concentration in Extract g/100 g of Extract (Chiarini et al. [7])	Concentration in Extract (TAN 1) g/100 g of Extract (Comandini et al. [9])	Concentration in Extract g/100 g of Extract (Present Work at Conditions of 150 °C, 35 min and 30 mL/g)
Vescalagin	2.31	4.08	0.91
Castalagin	2.26	3.80	1.56
1-*o*-galloyl-castalagin	/	3.20	0.13
Gallic acid	1.25	2.80	1.03
Ellagic acid	1.70	0.93	4.19

**Table 7:** The values of regression coefficients and analysis of the model for ellagic acid yield.

Parameter	Coefficient Estimate	Standard Error	Sum of Squares	Degrees of Freedom	Mean Square	*F*-Value	*p*-Value Probability > *F*
**Model**			101.37	5	20.27	35.97	<0.0001
**Intercept**	11.68	0.25		1			
**X_2_**	−1.18	0.27	11.16	1	11.16	19.81	0.0010
**X_3_**	3.07	0.27	75.40	1	75.40	133.78	<0.0001
**X_1_ X_2_**	−0.85	0.38	2.87	1	2.87	5.10	0.0453
**X_2_ X_3_**	0.87	0.38	2.99	1	2.99	5.31	0.0417
**X_2_^2^**	−1.45	0.36	8.94	1	8.94	15.87	0.0021
**Residual**			6.20	11	0.56		
**Lack of fit**			2.27	7	0.32	0.33	0.9042
**Pure error**			3.93	4	0.98		
***R*^2^**	0.9424		**Adj *R*^2^**	0.9162			
**C.V. %**	6.82		**Pred *R*^2^**	0.8711			
**PRESS**	13.86		**Adeq Precision**	21.613			

**Table 8:** Proposed experiments by the Box–Behnken method and the predicted and experimental values of sugar yields obtained by subcritical water extraction of chestnut bark.

Run	Temp. (X_1_), °C	Time (X_2_), min	Solvent-Solid Ratio (X_3_), mL/g	Glucose Yield, mg/g of Bark		Fructose Yield, mg/g of Bark		Maltose Yield, mg/g of Bark		Arabinose Yield, mg/g of Bark
				Experimental	Predicted	Experimental	Predicted	Experimental	Predicted	Experimental
1	150 (−1)	10 (−1)	20 (0)	13.2	13.3	13.0	14.5	17.9	18.1	9.2
2	250 (1)	10 (−1)	20 (0)	0	0.008	0	0.06	0	0.0004	0
3	150 (−1)	60 (1)	20 (0)	7.8	6.9	11.4	11.0	8.5	8.4	12.5
4	250 (1)	60 (1)	20 (0)	0	0.008	0	0.06	0	0.0004	0
5	150 (−1)	35 (0)	10 (−1)	9.5	10.6	13.9	12.7	11.2	11.4	16.5
6	250 (1)	35 (0)	10 (−1)	0	0.01	0	0	0	0.002	0
7	150 (−1)	35 (0)	30 (1)	9.3	9.2	12.5	12.7	14.7	14.3	11.4
8	250 (1)	35 (0)	30 (1)	0	0.01	0	0	0	0.002	0
9	200 (0)	10 (−1)	10 (−1)	15.3	14.1	14.9	13.4	5.9	5.1	5.9
10	200 (0)	60 (1)	10 (−1)	10.4	10.0	10.1	10.0	2.7	2.4	0
11	200 (0)	10 (−1)	30 (1)	12.8	12.5	13.8	13.4	5.9	5.8	9.7
12	200 (0)	60 (1)	30 (1)	7.5	8.6	6.8	10.0	3.0	2.9	0
13	200 (0)	35 (0)	20 (0)	11.7	11.2	13.4	11.6	4.2	3.9	0
14	200 (0)	35 (0)	20 (0)	11.8	11.2	12.5	11.6	3.6	3.9	0
15	200 (0)	35 (0)	20 (0)	10.8	11.2	12.7	11.6	3.7	3.9	0
16	200 (0)	35 (0)	20 (0)	11.5	11.2	12.0	11.6	4.0	3.9	0
17	200 (0)	35 (0)	20 (0)	10.0	11.2	9.6	11.6	2.9	3.9	0

**Table 9:** The values of regression coefficients and analysis of the models for yield of sugars.

GLUCOSE YIELD							
Parameter	Coefficient Estimate	Standard Error	Sum of Squares	Degrees of Freedom	Mean Square	*F*-Value	*p*-Value Probability > *F*
**Model**			34.11	5	6.82	350.71	<0.0001
**Intercept**	3.35	0.046		1			
**X_1_**	−1.57	0.049	19.72	1	19.72	1013.91	<0.0001
**X_2_**	−0.30	0.049	0.70	1	0.70	35.81	<0.0001
**X_3_**	−0.11	0.049	0.091	1	0.091	4.68	0.0533
**X_1_ X_2_**	0.21	0.070	0.18	1	0.18	9.08	0.0118
**X_1_^2^**	−1.78	0.068	13.42	1	13.42	690.07	<0.0001
**Residual**			0.21	11	0.019		
**Lack of fit**			0.16	7	0.023	1.75	0.3071
**Pure error**			0.053	4	0.013		
***R*^2^**	0.9938		**Adj *R*^2^**	0.9909			
**C.V. %**	5.55		**Pred *R*^2^**	0.9810			
**PRESS**	0.65		**Adeq Precision**	46.573			
**FRUCTOSE YIELD**							
**Parameter**	**Coefficient Estimate**	**Standard Error**	**Sum of Squares**	**Degrees of Freedom**	**Mean Square**	***F*-Value**	***p*-Value** **Probability > *F***
**Model**			37.10	3	12.37	212.08	<0.0001
**Intercept**	3.41	0.080		1			
**X_1_**	−1.78	0.085	25.37	1	25.37	435.04	<0.0001
**X_2_**	−0.25	0.085	0.51	1	0.51	8.78	0.0110
**X_1_^2^**	−1.63	0.12	11.22	1	11.22	192.43	<0.0001
**Residual**			0.76	13	0.058		
**Lack of fit**			0.57	9	0.063	1.34	0.4149
**Pure error**			0.19	4	0.047		
***R*^2^**	0.9800		**Adj *R*^2^**	0.9754			
**C.V. %**	9.14		**Pred *R*^2^**	0.9641			
**PRESS**	1.36		**Adeq Precision**	34.725			
**MALTOSE YIELD**							
**Parameter**	**Coefficient Estimate**	**Standard Error**	**Sum of Squares**	**Degrees of Freedom**	**Mean Square**	***F*-Value**	***p*-Value** **Probability > *F***
**Model**			27.33	6	4.56	338.9	<0.0001
**Intercept**	1.98	0.039		1			
**X_1_**	−1.79	0.041	25.68	1	25.68	1910.07	<0.0001
**X_2_**	−0.35	0.041	0.97	1	0.97	72.32	<0.0001
**X_3_**	0.077	0.041	0.047	1	0.047	3.50	0.0910
**X_1_ X_2_**	0.33	0.058	0.43	1	0.43	31.93	0.0002
**X_1_ X_3_**	−0.12	0.058	0.059	1	0.059	4.42	0.0619
**X_1_^2^**	−0.19	0.056	0.15	1	0.15	11.16	0.0075
**Residual**			0.13	10	0.013		
**Lack of fit**			0.067	6	0.011	0.67	0.6865
**Pure error**			0.067	4	0.017		
***R*^2^**	0.9951		**Adj *R*^2^**	0.9922			
**C.V. %**	6.13		**Pred *R*^2^**	0.9900			
**PRESS**	0.27		**Adeq Precision**	57.856			

**Table 10:** Proposed experiments by Box-Behnken method and the predicted and experimental values of sugar derivatives obtained by subcritical water extraction of chestnut bark.

Run	Temp. (X_1_), °C	Time (X_2_), min	Solvent-Solid Ratio (X_3_), mL/g	5-HMF Yield, mg/g of Bark		Furfural Yield, mg/g of Bark		Levulinic Acid Yield, mg/g of Bark
				Experimental	Predicted	Experimental	Predicted	Experimental
**1**	150 (−1)	10 (−1)	20 (0)	0.2	0.1	0.1	0.1	0
**2**	250 (1)	10 (−1)	20 (0)	8.0	6.8	9.5	7.8	18.6
**3**	150 (−1)	60 (1)	20 (0)	0.9	1.0	0.8	1.0	0
**4**	250 (1)	60 (1)	20 (0)	3.5	3.7	2.3	2.4	25.0
**5**	150 (−1)	35 (0)	10 (−1)	0.7	0.7	0.7	0.6	0
**6**	250 (1)	35 (0)	10 (−1)	5.1	5.2	4.0	4.0	22.3
**7**	150 (−1)	35 (0)	30 (1)	0.3	0.3	0.2	0.2	0
**8**	250 (1)	35 (0)	30 (1)	7.8	8.2	6.3	7.0	20.1
**9**	200 (0)	10 (−1)	10 (−1)	4.1	5.1	9.3	11.6	0
**10**	200 (0)	60 (1)	10 (−1)	11.0	10.1	21.6	20.3	20.2
**11**	200 (0)	10 (−1)	30 (1)	3.5	4.0	8.6	9.1	0
**12**	200 (0)	60 (1)	30 (1)	8.5	7.9	18.5	16.0	19.4
**13**	200 (0)	35 (0)	20 (0)	9.0	8.2	17.3	16.8	16.9
**14**	200 (0)	35 (0)	20 (0)	9.2	8.2	18.1	16.8	15.6
**15**	200 (0)	35 (0)	20 (0)	8.1	8.2	16.3	16.8	13.5
**16**	200 (0)	35 (0)	20 (0)	9.1	8.2	19.4	16.8	14.8
**17**	200 (0)	35 (0)	20 (0)	7.4	8.2	15.2	16.8	11.4

**Table 11:** The values of regression coefficients and analysis of the models for 5-HMF and furfural yields.

5-HMF YIELD							
Parameter	Coefficient Estimate	Standard Error	Sum of Squares	Degrees of Freedom	Mean Square	*F*-Value	*p*-Value Probability > *F*
**Model**			27.61	7	3.94	173.74	<0.0001
**Intercept**	2.10	0.060		1			
**X_1_**	1.32	0.053	13.88	1	13.88	611.54	<0.0001
**X_2_**	0.34	0.053	0.95	1	0.95	41.83	0.0001
**X_3_**	−0.12	0.053	0.11	1	0.11	4.98	0.0525
**X_1_ X_2_**	−0.64	0.075	1.63	1	1.63	71.83	<0.0001
**X_1_ X_3_**	0.35	0.075	0.48	1	0.48	21.18	0.0013
**X_1_^2^**	−1.55	0.073	10.09	1	10.09	444.33	<0.0001
**X_2_^2^**	−0.25	0.073	0.25	1	0.25	11.20	0.0086
**Residual**			0.20	9	0.023		
**Lack of fit**			0.17	5	0.034	3.91	0.1053
**Pure error**			0.035	4	8.670 × 10^−3^		
***R*^2^**	0.9927		**Adj *R*^2^**	0.9869			
**C.V. %**	11.97		**Pred *R*^2^**	0.9583			
**PRESS**	1.16		**Adeq Precision**	41.694			
**FURFURAL YIELD**							
**Parameter**	**Coefficient Estimate**	**Standard Error**	**Sum of Squares**	**Degrees of Freedom**	**Mean Square**	***F*-Value**	***p*-Value** **Probability > *F***
**Model**			44.65	7	6.38	281.09	<0.0001
**Intercept**	2.82	0.060		1			
**X_1_**	1.32	0.053	14.04	1	14.04	618.64	<0.0001
**X_2_**	0.28	0.053	0.63	1	0.63	27.65	0.0005
**X_3_**	−0.12	0.053	0.11	1	0.11	5.01	0.0520
**X_1_ X_2_**	−0.87	0.075	3.03	1	3.03	133.69	<0.0001
**X_1_ X_3_**	0.40	0.075	0.65	1	0.65	28.61	0.0005
**X_1_^2^**	−2.47	0.073	25.69	1	25.69	1131.99	<0.0001
**X_2_^2^**	−0.21	0.073	0.18	1	0.18	7.93	0.0202
**Residual**			0.20	9	0.023		
**Lack of fit**			0.17	5	0.034	3.83	0.1088
**Pure error**			0.035	4	8.823 × 10^−3^		
***R*^2^**	0.9954		**Adj *R*^2^**	0.9919			
**C.V. %**	9.61		**Pred *R*^2^**	0.9733			
**PRESS**	1.20		**Adeq Precision**	51.689			
